# Pathology and Treatment of Some Forms of Headache

**Published:** 1884-05

**Authors:** 


### Pathology and Treatment of Some Forms of Headache.

At a meeting of the medical society of Islington, last week, a very interesting communication was read by Dr. T. Lauder Brunton, F. R. S., on this subject, of which the main points were as follows: Headache is usually the product of two factors—local irritation and general condition. The chief local causes are decayed teeth and abnormalities of the eye, although disease of the ear and nose, inflammation of the throat, and local irritation of the pericranium, or of the skull in rheumatism and syphilis, are not to be forgotten. Decayed teeth may give rise to temporal or occipital headache when the molars are affected, and also, I think, the frontal when the incisors are decayed. The chief abnormal conditions of the eye are strain from reading, or working with imperfect light, or for too long a time, myopia, hypermetropia, astigmatism, and inequality of vision between the two eyes. Besides these, I think that alterations in the circulation and intraocular pressure are frequently produced by bile or poisonous substances circulating in the blood, and that probably also a rheumatic condition affecting either the eye itself or the muscles which move it is a not uncommon source of headache. Where both eyes are equally affected the headache is usually frontal, but when one eye is more affected than the other the headache appears either in the form of brow ague or megrim. In treating any case of headache, therefore, the first thing to do is to see whether the teeth are sound and the eyes normal. If anything is found wrong with either the teeth or the eyes, the defect should be at once corrected. The throat, ears and nose should be examined to see if any source of irritation is present there, and the surface of the scalp tested by pressure for rheumatic or syphilitic inflammation. The locality of headache is probably determined chiefly by the local source of irritation, but this differs according to the general condition. Thus frontal headache with constipation is usually relieved by purgatives; frontal headache just above the eyebrows, without constipation, is relieved by acid; and a similar headache, situated higher up, at the commencement of the hairy scalp, is relieved by alkalies. Vertical headache is usually associated with anemia, and is relieved by iron. The more or less continuous headache of syphilis is usually best relieved by iodide of potassium, but in order to gain relief the dose must sometimes be much larger than that usually given, and may range from five grains up to thirty grains for a dose. Similar quantities of iodide of potassium are usually sufficient to cure the rheumatic headache.—*Louisville Medical News.*

